# Neural tension patterns during cervical spine rotation: diagnostic implications from a cadaveric study

**DOI:** 10.1186/s12998-025-00608-w

**Published:** 2025-10-09

**Authors:** Daniel Alvarez, Rob Sillevis, Juan Nicolás Cuenca Zaldívar, Eleuterio A. Sánchez Romero

**Affiliations:** 1 Department of Rehabilitation Sciences, Florida Gulf Coast University, Fort Myers, FL 33965 USA; 2 Departamento de Enfermería y Fisioterapia, Facultad de Medicina y Ciencias de la Salud, Universidad de Alcalá, Grupo de Investigación en Fisioterapia y Dolor, Madrid, Spain; 3 Physical Therapy Unit, Primary Health Care Center “El Abajón”, Las Rozas de Madrid, Spain; 4 Research Group in Nursing and Health Care, Puerta de Hierro Health Research Institute-Segovia deArana (IDIPHISA), Majadahonda, Spain; 5 Interdisciplinary Research Group on Musculoskeletal Disorders, Madrid, Spain; 6 Physiotherapy and Orofacial Pain Working Group, Sociedad Española de Disfunción Craneomandibular y DolorOrofacial (SEDCYDO), 28009 Madrid, Spain

**Keywords:** Dissection, Neural tension, Cervical spine, Cadaver study, Occipital neuralgia, Flexion-rotation test, Upper limb tension test

## Abstract

**Background:**

Cervical neural tension reflects the biomechanical and physiological responses of spinal nerves to positional changes. Although clinical tests exist for the lower cervical spinal nerve, tension patterns in the upper and mid-cervical nerves remain underexplored, limiting the diagnostic accuracy for conditions such as occipital neuralgia.

**Methods:**

This cadaveric study quantified tensile load changes at the cervical spinal nerve level (C1–C5) during passive cervical spine rotation in five formalin-embalmed cadavers. Tension was measured on the cervical spinal nerves (C1–C5) using force gauges attached proximal to the division between the dorsal and ventral rami. C1 measurements were obtained from a single specimen. Two movement conditions were used: cervical flexion-rotation for C1–C3 and neutral-plane rotation for C4–C5.

**Results:**

Ipsilateral increases in neural tension were observed in C1–C3 during flexion-rotation movements. By contrast, C4–C5 exhibited a consistent pattern of contralateral load increase during rotation in the neutral plane. Statistically significant variations in the tensile load were observed at the C5 level under different rotation conditions, specifically at C5 left (*p* = 0.003) and C5 right (*p* = 0.006). Post-hoc analyses of C5 measurements during neutral-plane rotation revealed significant differences between right and left rotation (*p* = 0.018) and between left rotation and neutral rotation (*p* = 0.018) on the left side, as well as between right rotation and left rotation and neutral rotation (*p* = 0.026, *p* = 0.024) on the right side. Intraclass correlation coefficients (ICC) indicated good-to-excellent reliability (ICC > 0.75), particularly at C2–C5.

**Conclusions:**

Cervical rotation influenced neural tension, with distinct patterns observed between the upper cervical segments (tested under flexion-rotation) and the middle cervical segments (tested under neutral plane rotation). These exploratory findings suggest that replacing lateral neck flexion with rotation in the upper-limb tension test may represent a promising direction for future research. Additionally, the flexion-rotation test may provide a basis for clinical validation as a potential indicator of greater occipital nerve tension. These results lay the groundwork for refining neurodynamic assessments and warrant further in vivo investigation.

## Introduction

Neural tension can be defined as the mechanical state of a nerve resulting from the application of tensile or compressive forces, reflecting the dynamic interaction between the nervous system and surrounding musculoskeletal structures. Nerves are located in and between the muscles and fascia and within specific tunnels in the skeletal system. If nerves do not have the proper amount of movement, blood flow, and space, they are unable to tolerate body movement, resulting in decreased nerve function and symptoms such as numbness, tingling, burning, or pain [1]. Neural tension has also been referred to in the literature as a neurodynamic dysfunction or adverse neural tension. It is a direct reflection of tensile and compressive mechanical loading and of the physiological responses of the nervous system to movement [2, 3]. Increased neural tension implies that nerves cannot slide and glide through tissues optimally [4]. The spinal nerve appears to be more sensitive to tensile load than the peripheral nerves because of its limited connective tissue support and higher vulnerability to vascular compromise under tension [5]. Tensile loads of 5–10% of maximal load tolerance have been demonstrated to result in alterations in blood flow in the epineural and perineural vessels [6]. Such vascular changes can result in neural dysfunction, mechanosensitivity, and pain during movement or sustained postures [7].

Abnormal cervical neural tension is a clinical phenomenon with an estimated prevalence of approximately 1.5–5.8% in the general population, as reported in systematic reviews of cervical radiculopathy epidemiology [8]. Clinically, neural tensile tolerance is assessed using tests such as the upper limb tension test (ULLT) of the lower cervical spinal nerve [8, 9]. However, there is a high prevalence of positive ULTT results, even in asymptomatic individuals, with positive findings reported in as many as 80–90% of cases, depending on the nerve and criteria used [9]. Currently, there are no reports on the quantification or elicitation of neural tension in the upper three cervical nerves. This lack of evidence might explain the diagnostic and epidemiological conflicts observed in the literature for cranial neuralgias, such as occipital neuralgia and cervicogenic headaches, as well as the limited reliability of cervical neurodynamic assessments reported in recent reviews of cervical radiculopathy [10, 11].

Unlike the peripheral nerves and their rami, the cervical spinal nerve lacks the capacity for significant sliding or gliding within the surrounding tissues. Their position within the vertebral canal and limited connective tissue support make them more vulnerable to tensile loading and vascular compromise, which may contribute to mechanosensitivity and pain [1]. Abnormal tolerance to tensile neural tensioning can manifest as pain, restricted range of motion, altered sensation, and functional impairment [7].

Human nerves exhibit minimal elastic elongation (up to 6%, without damage) [12]. In contrast, fascia-containing structures demonstrate adaptive lengthening based on mechanical demands and position over time, which limits their active range of motion [13, 14]. Forward head posture is an example in which chronic myofascial adaptations and increased cervical curvature may alter the angulation of nerve pathways, resulting in indirect tensile loading of the suboccipital nerve structures despite reduced linear distance [15]. This effect may particularly influence the greater occipital nerve and dorsal ramus of C2 due to their anatomical course and fascial attachment [15–17].

The upright position results in a vertical gravitational compressive force on the cervical spine due to the weight of the head [15]. While the long-term consequences of such loading remain uncertain, it is plausible that, when combined with postural deviations such as increased cervical lordosis or posterior head rotation, these forces may increase the mechanical demands on cervical structures [15, 16]. Such biomechanical alterations could influence the cervical spinal nerve, which innervates the prevertebral muscles, trapezius, sternocleidomastoid, atlantoaxial joint capsules, upper cervical stabilizing ligaments, and dura mater and may thereby contribute to conditions such as cervicogenic headache or occipital neuralgia [10, 15]. The trapezius and sternocleidomastoid muscles are primarily innervated by the accessory nerve (cranial nerve XI), with proprioceptive contributions from the cervical spinal nerves, reflecting the complex anatomical and functional interplay in this region [15–17].

Cervical motion, especially upper cervical rotational movement, influences the orientation of the spinal cord within the spinal canal, suggesting tension changes in the spinal nerves with passive cervical motion [15]. Sillevis et al. [15] identified that passive high cervical rotation resulted in ipsilateral translation of the spinal cord in the transverse plane, suggesting a decrease in tension in the nerves of the upper cervical spine on the side of rotation. Previously, Ranger et al. identified similar translational movements of the spinal cord in the lumbar spine were identified by Ranger et al. [16] during positional changes.

It has been suggested that the collagenous connections between the nuchal ligament and the dura, and the suboccipital muscles and the dura through the myodural bridges prevent compression of the cord during cervical movement [17]. Additionally, fibrous fascial connections anchor the dura to the ligamentum flavum or the lamina and the anterior aspect of the dura to the posterior longitudinal ligament [18]. Meningovertebral ligaments have been described in the literature as extending along the length of the vertebral column [18], forming structural connections between the dura mater and vertebral elements. Based on the presence of myodural bridges, a posterior-directed pull of the dura can be expected with active suboccipital contraction during upper cervical extension, side bending, and rotational movements. Furthermore, passive movement of the head and cervical spine creates tension in the meningovertebral ligaments and increases the neural tension in the cervical spinal nerves [19–21]. Therefore, it can be postulated that the position of the upper cervical spine and head impacts tension on the dura, thus limiting the ability of the peripheral nerve to withstand tensile force [17, 22]. This construct is further supported by the findings of Antolinos-Campillo et al. [23], who demonstrated a reduction in median nerve neural tension after suboccipital muscle inhibition. Additionally, considering the location and possible fascial connections between the greater and lesser occipital nerves and cervical region structures, there might be a correlation between suboccipital nerve tension and upper cervical positioning and movement. Upper cervical spine movements may contribute to tensile loading of the greater and lesser occipital nerves, potentially causing neural tension and pain [18, 24]. Such neural tension has been identified by clinicians when treating patients with headaches [19–21].

Cadaveric studies have been widely used to explore these mechanisms and provide valuable insights into the cervical neuroanatomy and biomechanics. For instance, Scali et al. [17] described myodural bridges linking the suboccipital musculature with the dura mater, whereas Enix et al. [25] reviewed cadaveric evidence of cervical myodural connections and their clinical implications. Such studies highlight the utility of cadaver models in advancing our understanding of neurodynamic phenomena and justify the present study.

This exploratory cadaver study aimed to quantify the tensile load experienced by cervical spinal nerves close to their exit from the intervertebral foramen during cervical rotation. These exploratory data may help address diagnostic uncertainties and provide a preliminary biomechanical basis for future studies aimed at refining the clinical application of neural tension techniques related to cervical rotation, but they cannot be directly translated into clinical practice.

## Materials and methods

### Population

**Fig. 1:**
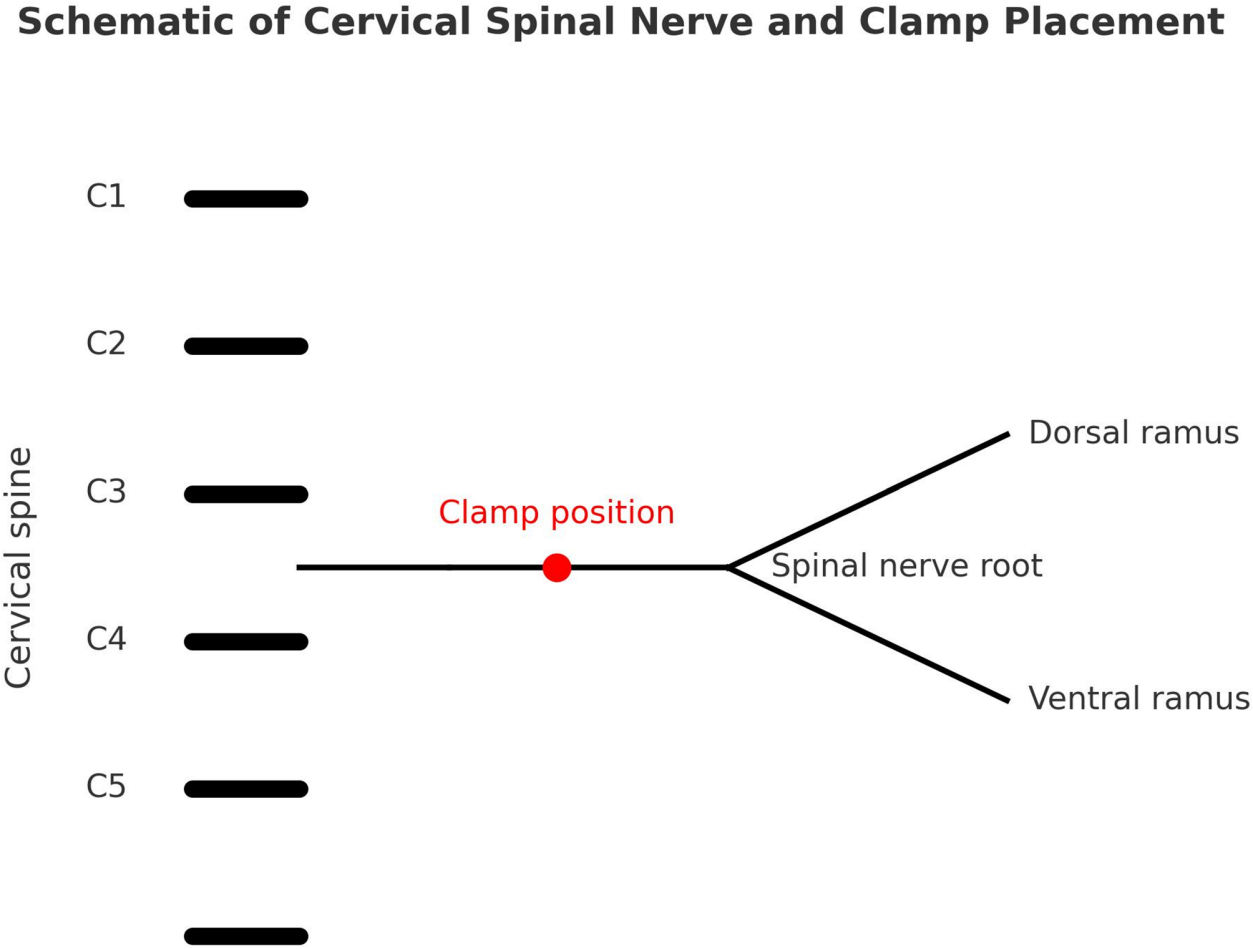
Anatomical schematic of cervical spinal nerve and clamp placement. Schematic illustration of a cervical spinal nerve root exiting the intervertebral foramen and dividing into dorsal and ventral rami. The red dot indicates the approximate clamp position used to attach the force gauge clamp in this study

Seven adult cadavers (aged 54–82 years) were included in this study. However, owing to anatomical limitations, tissue degradation, or intra-dissection nerve damage, only five cadavers provided usable nerve structures for data collection. These five specimens were used for all final measurements and analyses. Cadavers were obtained from the Anatomical Board of the State of Florida following the Anatomical Board regulations and Florida State Law. All cadavers were embalmed using 10% formalin solution, which is the standard concentration for anatomical preservation in cadaveric studies. The study design and methodological reporting followed the QUACS guidelines for cadaveric studies, proposed by Wilke et al. [26]. No medical history of cadavers was reported. Students at the Florida Gulf Coast University’s Doctor of Physical Therapy had previously dissected their cadavers. To illustrate the anatomical context and experimental procedure, Fig. 1 presents a schematic representation of the cervical spinal nerve exiting the intervertebral foramen and its division into the dorsal and ventral rami. The diagram also indicates the approximate clamp position used in this study for tensile load measurement, which is marked by a red dot.

**Fig. 2:**
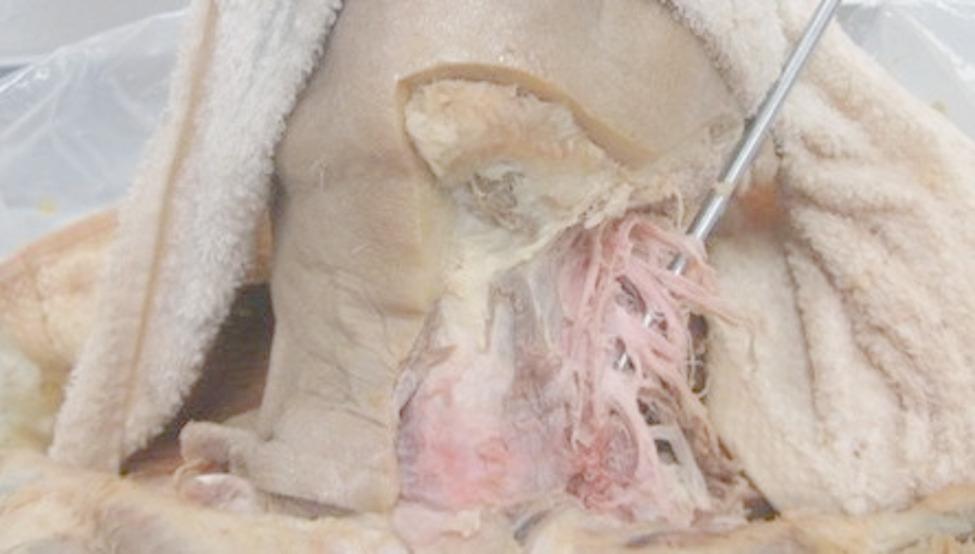
Exposure of the cervical plexus

The dissection steps for exposing the cervical plexus followed established anatomical protocols [17, 26], ensuring methodological consistency. After removal of the skin and superficial fascia, the sternocleidomastoid muscle was retracted from the sternal attachment and the carotid artery and jugular vein were carefully dissected from the clavicle to the cranium. These procedures provide access to the deeper neurovascular structures. The cervical plexus was then fully exposed and its components were separated on both sides for subsequent measurements (Fig. 2).

### Measurement set up

**Fig. 3:**
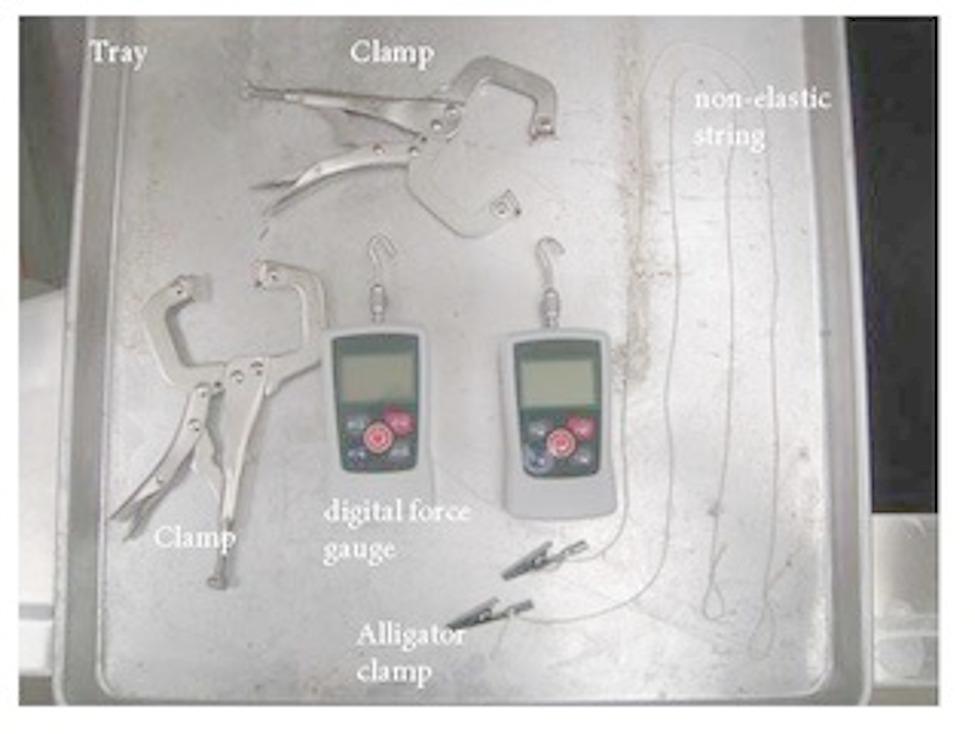
Measurement tools

The tools used to measure neural tension included digital force gauges, alligator clamps, non-elastic strings, clamps, and a tray (Fig. 3).

The figure shows the experimental equipment used in the study: digital force gauge, alligator clamp, clamp, non-elastic string, and tray.

The digital force gauges (VTSYIQI, 500 N capacity) used in this study had a manufacturer-reported full-scale measurement tolerance of ± 0.3%, corresponding to a maximum error margin of ± 1.5 N.

**Fig. 4:**
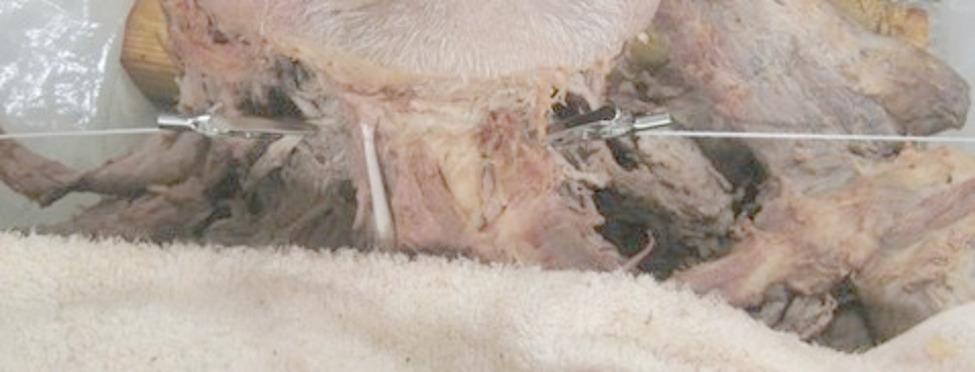
Clamp attachment on the cervical spinal nerve. The alligator clamp was positioned ~ 5 mm distal to the intervertebral foramen on the cervical spinal nerve (mixed nerve), before its division into the dorsal and ventral rami (not on a root or on a ramus). The image has been cropped to remove non-essential elements

They were set up by tying alligator clamps to one end of the string, hooking them to the force gauge at the other end of the string, and placing them on the baking tray. A stool was placed next to the cadaver bed to allow the force gauges to be attached to the cervical plexus. Adjustments were made with a wooden block to increase the stool height. The alligator clamp was attached approximately 5 mm from the intervertebral foramen to the cervical spinal nerve (mixed nerve) after the dorsal and ventral cervical spinal nerves had joined and before division into the dorsal and ventral rami. This ensured consistent placement at the spinal nerve level (rather than on the nerve root or rami) across specimens, thereby maximizing the anatomical accuracy and reproducibility (Fig. 4).

In this study, a detectable change was defined as any variation in tensile force exceeding ± 1.5 N, based on the measurement tolerance of the digital force gauges. Changes below this threshold were considered within the margin of instrument error and were not interpreted as true variations in neural tension.

The force gauge was pulled away until it reached an arbitrary positive number, and secured in place using clamps to detect any loss of tension. The apparatus was assembled by securing alligator clamps to one end of the string, connecting the opposite end to the force gauge, and positioning the entire setup on a tray supported by a stool to stabilize the force gauges during testing.

An arbitrary positive number, typically around 5 Newtons, was chosen as a baseline force to strike a balance between avoiding nerve damage and ensuring detectable changes. This value was deemed sufficient for capturing tension variations while minimizing the risk of harm to the nerves, and allowed for standardization.

### Measurement protocol

To provide a clear overview of the anatomical availability of cervical nerves across specimens, we have summarized this information in Table 1. This table specifies which cervical levels were accessible for measurement and whether they were bilaterally available.

**Table 1:** Availability of cervical spinal nerves across cadavers (nerves accessible at each level and specimen count)

Cervical level	Number of cadavers available (*n*)	Notes
C1	1	Only one specimen preserved C1 nerves suitable for measurement
C2	2	Two cadavers had intact C2 nerves available
C3	5	Present bilaterally in all cadavers
C4	5	Present bilaterally in all cadavers
C5	5	Present bilaterally in all cadavers

This table summarizes the number of cadavers in which each cervical spinal nerve (C1–C5) was available for tensile load measurements

For all levels, the tensile load was recorded from the cervical spinal nerve segment described above; no measurements were taken from the dorsal/ventral roots or from the rami.

Testing began once all set-up processes had been established, and the gauges were set at a nearly equal value of approximately 5 Newtons on the available uppermost set of cervical nerves. All cadavers had nerves C3–C5 available on both sides, two cadavers had nerves available at the C2 level, and only one cadaver with C1 nerves was available for testing. A secure grip was obtained on the head of the cadaver, and the head was brought to full flexion such that only suboccipital rotation occurred.

Flexion rotation was selected for C1–C3 because this maneuver preferentially loads the upper cervical segments and isolates their contribution by limiting mid- and lower-cervical motions. In contrast, C4–C5 were tested under neutral-plane rotation to reflect their typical kinematics at the mid-cervical level and to avoid confounding effects from the upper cervical coupling patterns. This rationale was included to enhance the clarity of non-specialist readers.

A secure manual grip was chosen to allow precise control of both flexion and rotational movements, while minimizing unintended lateral flexion or axial compression. This method provided greater tactile feedback and adaptability to anatomical variations across specimens, ensuring consistent positioning throughout the trials. Baseline values were documented using force gauges, and the head was rotated to the right. Each nerve level was tested twice using identical procedures in both trials, including the same head positioning sequence, baseline recording, timing of 5 s for tissue settling, and subsequent tension measurement. These repeated trials aimed to ensure consistency and assess the intra-observer reproducibility.

It is important for researchers to focus on preventing lateral flexion in either direction as this could skew the results. After holding the rotation for 5 s, the tissues were settled and the forces on both gauges were documented. The researcher returned the cervical spine to the neutral rotation position while maintaining full flexion, re-recorded baseline scores, and turned it to the left. The values from the force gauges were recorded after 5 s of the left rotation. After returning to neutral condition, the process was repeated in the second trial. After the second trial, the cadaver’s head was released and the clamps were moved to the following pair of nerves. Once the fourth cervical nerve is reached, the researcher no longer brings the cervical spine into full flexion. Full flexion was deemed unnecessary as the focus shifted from the suboccipital to full cervical rotation. Consequently, only the rotation in the neutral transverse plane, which is reflective of the cadaver’s resting posture, was considered in further trials.

To assess intra-observer reliability, each nerve level and movement condition was measured twice using identical procedures by the same investigator. This approach minimized the variability associated with multiple raters and ensured methodological consistency. Reliability was quantified using intraclass correlation coefficient (ICC), Standard Error of Measurement (SEM), and Minimal Detectable Change (MDC). Inter-rater reliability was not assessed in this study because all dissections and measurements were performed by a single examiner to preserve the consistency across specimens.

To standardize cervical motion, the head was rotated passively to the anatomical end range of motion in both directions, defined by the first point of tissue resistance, without overpressure. This ensured comparable testing conditions across specimens, while acknowledging that natural anatomical asymmetry may have led to slight differences in the degree of rotation between the sides.

### Statistical analysis

For statistical analysis, R Ver. 4.1.3 (R Foundation for Statistical Computing, Institute for Statistics and Mathematics, Welthandelsplatz 1, 1020 Vienna, Austria). The significance level was set at *P* < 0.05. Variables are described as median (interquartile range).

Based on the three movement conditions (right rotation, left rotation, and neutral), the intra-observer two-way mixed single measures (consistency/absolute agreement) Intraclass Correlation Coefficient (ICC) (3, 1) was calculated as relative reliability, defining poor (< 0.5), moderate (0.5–0.75), good (0.75–0.9), and excellent (>0.9). Absolute reliability was determined using standard error of measurement (SEM). The minimum detectable change (MDC) was calculated.

The presence of significant differences in neural tension between the left and right rotation positions and the neutral position was analyzed using a robust permutation test owing to the small sample size [27], with Bonferroni correction applied in post-hoc tests.

## Results

Good or excellent intraobserver reliability was observed in measurements of the left C2 and C3 cervical spinal nerves during left rotation trials, in C4 on both sides during left rotation, and in left C5 during right rotation (Table 2).

**Table 2:** Reliability of tensile load measurements

	ICC (95%CI)	Mean (95%CI)	SEM (95%CI)	MDC
*C2 left*				
Right rotation	**0.937 (0.328, 1)**	5.133	0.321	0.891
Left rotation	**0.793 (− 0.122, 0.999)**	6.967	1.893	5.247
Neutral	0.282 (− 0.089, 0.992)	4.933	1.3	3.604
*C2 right*				
Right rotation	0 (− 0.461, 0.985)	5.667	2.15	5.960
Left rotation	0 (− 0.461, 0.985)	6.667	3.055	8.468
Neutral	0.553 (0.044, 0.997)	4.833	1.125	3.120
*C3 left*				
Right rotation	**0.873 (0.607, 0.977)**	6.75 (− 9.98, 23.48)	1.433 (− 4.105, 6.971)	3.972
Left rotation	0.269 (− 0.177, 0.796)	6.633	2.916	8.083
Neutral	0 (− 0.122, 0.445)	5.358 (4.829, 5.888)	1.192 (− 2.886, 5.27)	3.304
*C3 right*				
Right rotation	0.492 (0.006, 0.883)	5.533 (− 10.985, 22.051)	1.387 (− 7.073, 9.847)	3.844
Left rotation	0.244 (− 0.194, 0.784)	7.733 (− 0.314, 15.781)	1.538 (0.726, 2.351)	4.264
Neutral	0 (− 0.122, 0.445)	6.517 (6.517, 6.517)	0.749 (− 1.409, 2.908)	2.077
*C4 left*				
Right rotation	0.628 (0.162, 0.922)	4.583 (− 10.452, 19.619)	0.554 (− 3.746, 4.853)	1.535
Left rotation	**0.814 (0.471, 0.966)**	2.083 (− 4.058, 8.225)	0.981 (− 7.456, 9.417)	2.718
Neutral	0.593 (0.286, 0.903)	3.342 (− 3.753, 10.436)	0.158 (0.03, 0.286)	0.437
*C4 right*				
Right rotation	0.537 (0.053, 0.897)	3.45 (− 6.503, 13.403)	0.737 (− 5.509, 6.983)	2.043
Left rotation	**0.898 (0.672, 0.982)**	6.333 (− 21.197, 33.863)	1.018 (− 3.46, 5.496)	2.822
Neutral	0.589 (0.281, 0.901)	3.925 (− 6.981, 14.831)	0.364 (− 1.315, 2.042)	1.008
*C5 left*				
Right rotation	**0.757 (0.361, 0.953)**	6.389 (3.882, 8.895)	0.643 (− 0.16, 1.445)	1.782
Left rotation	0.231 (− 0.202, 0.778)	2.911 (1.751, 4.071)	0.392 (− 0.363, 1.146)	1.085
Neutral	0.189 (− 0.028, 0.683)	4.833 (3.952, 5.715)	0.99 (− 0.415, 2.396)	2.745
*C5 right*				
Right rotation	0 (− 0.327, 0.627)	2.922 (2.444, 3.4)	0.624 (− 0.108, 1.355)	1.729
Left rotation	0 (− 0.327, 0.627)	5.378 (3.846, 6.91)	2.245 (− 2.412, 6.902)	6.222
Neutral	0.33 (0.059, 0.785)	4.311 (2.894, 5.729)	0.996 (0.151, 1.84)	2.760

Reliability outcomes across cervical levels and movements. ICC, intraclass correlation coefficient; SEM, standard error of measurement; MDC, minimal detectable change; 95%CI, 95% confidence interval. ICC values higher than 0.75 are shown in bold

Table 3 presents the tensile load measurements recorded at each cervical level (C1–C5) under right rotation, left rotation, and neutral conditions. Although variability was noted across levels, a consistent trend of contralateral load increase was observed in C4 and C5 during the rotation. At the upper cervical levels (C1–C3), there was a general tendency for the ipsilateral tension to increase, although this did not reach statistical significance. These findings support the hypothesis that cervical rotation induces level-specific mechanical responses in spinal nerves. It should be noted that the values for C1 represent data from a single specimen and thus lack variability estimates.

**Table 3:** Neural tension by vertebral level

	Right rotation	Left rotation	Neutral	^a^*p* value
C1 left (N)	4.85	4.65	4.02	0.368
C1 right (N)	3.90	4.45	3.98	0.368
C2 left (N)	4.77 [4.58, 4.95]	4.71 [3.58, 5.84]	4.29 [3.97, 4.61]	0.867
C2 right (N)	4.91 [4.53, 5.29]	6.43 [6.32, 6.55]	4.05 [3.66, 4.44]	0.156
C3 left (N)	4.75 [3.80, 5.43]	6.35 [5.35, 6.63]	5.40 [5.32, 5.40]	0.796
C3 right (N)	5.40 [4.23, 6.60]	5.95 [4.60, 7.10]	6.45 [6.08, 6.52]	0.463
C4 left (N)	5.10 [3.55, 5.77]	2.57 [2.55, 3.65]	4.40 [3.90, 4.45]	0.127
C4 right (N)	3.00 [2.90, 3.60]	6.45 [4.17, 8.50]	4.50 [4.38, 4.78]	0.051
C5 left (N)	6.23 [5.47, 7.10]	3.15 [2.77, 3.30]	4.77 [4.52, 5.22]	**0.003**
C5 right (N)	3.03 [2.90, 3.03]	6.00 [5.37, 6.30]	4.30 [4.23, 4.92]	**0.006**

Statistical significance of neural tension variations at C5. “Neutral” refers to the tension measured during rotation in the neutral transverse plane, not to baseline values prior to movement initiation. Data expressed with median [interquartile range]. ^a^significant if *p* < 0.05 (shown in bold) for omnibus comparison between overall three tests. Specimens per level: C1 = 1; C2 = 2; C3–C5 = 5. Values for C1 reflect a single measurement and are presented without statistical variability

**Fig. 5:**
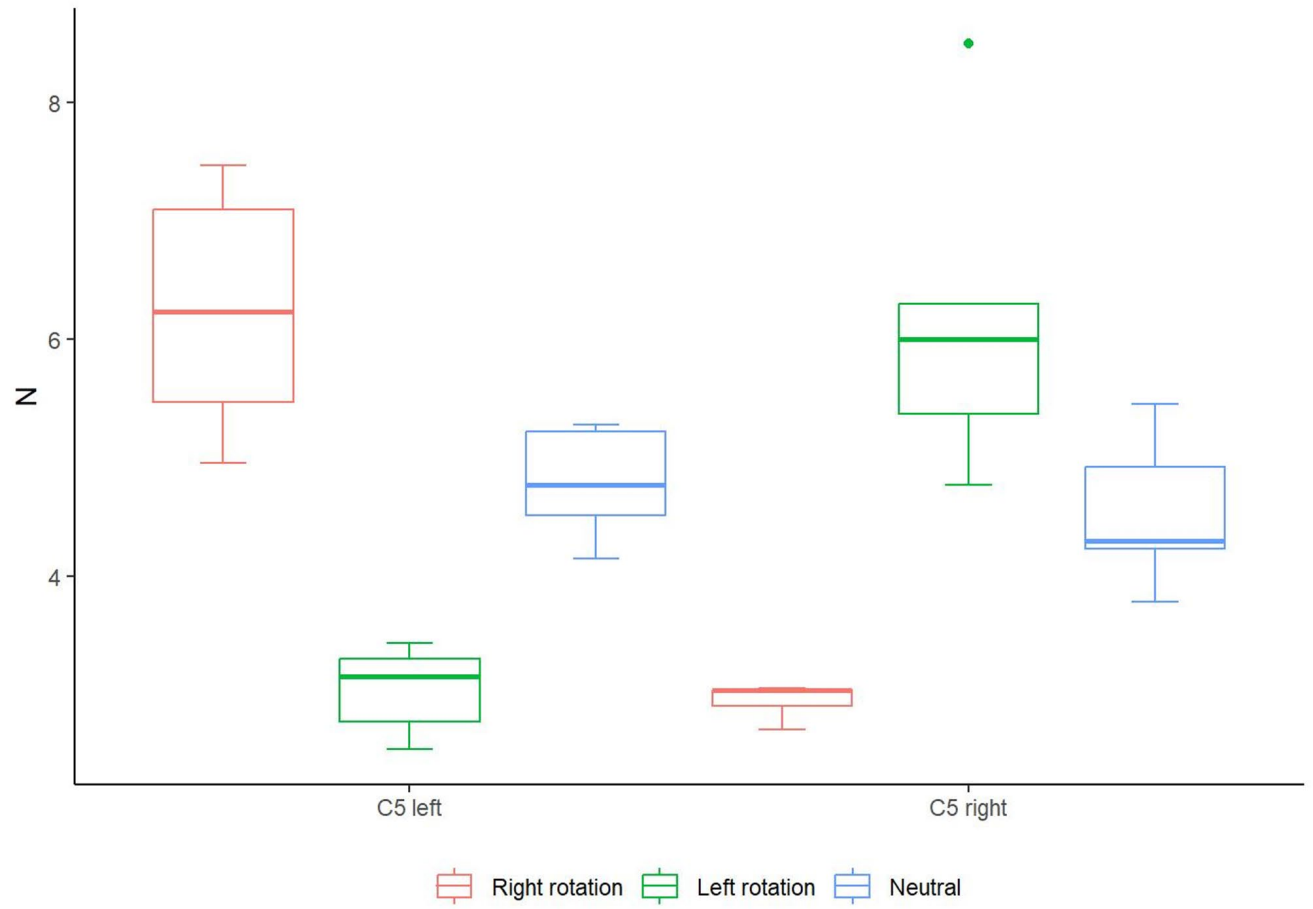
Box plots of vertebral levels showing significant neural tension differences

In this analysis, neutral denotes the tension values measured during head rotation in the neutral transverse plane, reflecting the resting posture condition. Post-hoc tests showed the presence of significant differences in C5 left between the tested movements of right rotation and left rotation (*p* = 0.018), with higher values in right rotation, and between left rotation and neutral rotation (*p* = 0.018), with higher values in neutral rotation. Significant differences were also observed in C5 right between the tested movements, right rotation-left rotation, and right rotation-neutral (*p* = 0.026, 0.024 respectively), with lower values in right rotation in both comparisons (Table 4; Fig. 5). The values shown in Fig. 5 correspond to the raw tensile force readings recorded during each movement condition, and were not adjusted relative to the initial baseline force.

**Table 4:** Post-hoc pairwise comparisons of neural tension across cervical levels

	^a^*p* value
*C1 left*	
Right rotation–Left rotation	0.952
Right rotation–Neutral	0.952
Left rotation–Neutral	0.952
*C1 right*	
Right rotation–Left rotation	0.952
Right rotation–Neutral	0.952
Left rotation–Neutral	0.952
*C2 left*	
Right rotation–Left rotation	> 0.999
Right rotation–Neutral	> 0.999
Left rotation–Neutral	> 0.999
*C2 right*	
Right rotation–Left rotation	0.489
Right rotation–Neutral	> 0.999
Left rotation–Neutral	0.357
*C3 left*	
Right rotation–Left rotation	> 0.999
Right rotation–Neutral	> 0.999
Left rotation–Neutral	> 0.999
*C3 right*	
Right rotation–Left rotation	> 0.999
Right rotation–Neutral	0.562
Left rotation–Neutral	> 0.999
*C4 left*	
Right rotation–Left rotation	0.262
Right rotation–Neutral	0.754
Left rotation–Neutral	0.674
*C4 right*	
Right rotation–Left rotation	0.145
Right rotation–Neutral	0.155
Left rotation–Neutral	0.416
*C5 left*	
Right rotation–Left rotation	**0.018**
Right rotation–Neutral	0.104
Left rotation–Neutral	**0.018**
*C5 right*	
Right rotation–Left rotation	**0.026**
Right rotation–Neutral	**0.024**
Left rotation–Neutral	0.164

Pairwise comparisons of neural tension under right rotation, left rotation, and neutral transverse plane rotation are presented for each cervical level. “Neutral” refers to head rotation performed in the neutral transverse plane, without cervical flexion. ^a^significant if *p* < 0.05 (shown in bold) in each pair of tests compared at each vertebral level

## Discussion

Our exploratory findings suggest that cervical spine rotation may influence neural tension patterns, particularly at the mid-cervical levels; however, these observations should be interpreted with caution and considered as preliminary contributions to the existing knowledge rather than definitive conclusions.

The findings of this study highlight the influence of cervical spine rotation on neural tension patterns, particularly at C4–C5 levels. At the C4–C5 levels, changes in tensile load appeared to vary consistently with the head position. Post hoc analyses indicated differences between rotation conditions, supporting the possibility that cervical rotation plays a more important role than previously assumed in neural tension assessment. Although normative in vivo values for cervical spinal nerve tension are lacking, the recorded changes (2–6 N) fall within the ranges shown in cadaveric and in vitro models to influence neural tissue fluid dynamics and mechanosensitivity [28, 29]. These magnitudes exceed the minimum detectable change (MDC) established for our measurement system and likely reflect physiologically meaningful variations. These findings are broadly consistent with prior observations of cervical biomechanics and neural tissue loading, such as ipsilateral translation of the spinal cord during upper cervical rotation (Sillevis & Hogg, 2020) [15] and positional shifts of the spinal cord observed by Ranger et al. (2008) [16].

A key observation was that right rotation increased tension in the left C5 cervical spinal nerve, whereas left rotation resulted in greater tension in the right side. Post-hoc comparisons indicated differences between rotation conditions, suggesting that cervical rotation may contribute more substantially to neural tension assessments than previously recognized. Although statistical significance was observed, the clinical relevance of these findings warrants further in vivo investigation.

The clinical implications of these results are relevant to neural tension testing. Our study suggests that modifying the Upper Limb Tension Test (ULLT) to incorporate cervical rotation rather than lateral flexion may provide a more accurate representation of nerve load. However, given the cadaveric nature of this study, these findings must be validated in live subjects, where muscle activity and spinal stabilizing mechanisms may influence neural behavior. Future studies should assess whether similar tension patterns occur under dynamic weight bearing conditions.

The observed trends in neural tension across different conditions align with the mechanical interpretation proposed in our analysis. The inclusion of such data visualization strengthens our interpretation of the results and provides clinicians with a reference for understanding the mechanical effects of head positioning.

Cadaveric studies offer unique advantages such as direct anatomical access, the ability to control positioning and load application, and the repeatability of mechanical testing protocols under fixed conditions. However, substantial variability may still occur across specimens, even when donors are matched for age, sex, and body size [26, 30].

Nerves of the upper cervical spine (C2–C3) contribute to the occipital nerve. Increased neural tension of the occipital nerves has been associated with conditions such as occipital neuralgia and cervicogenic headaches [10]. The observed changes in the tensile load of the upper cervical spinal nerves (C1–C3) in this study exhibited a tendency toward increased spinal nerve tension on the side of the rotation. Although the consistency varied, most trials displayed an increase in the tensile load. This finding correlates with the direct in vitro observation of Sillevis and Hogg [15], who identified that the spinal cord translated in the transverse plane to the ipsilateral side during the axial rotation of the atlas. Biomechanically, this can be explained by the fact that during passive left rotation, the left lateral mass of the atlas translates posteriorly, increasing ipsilateral nerve tension and accounting for the observed change in cord position [31]. Therefore, one could hypothesize that the results of both in vitro studies support the hypothesis that the translatory shift of the spinal cord is the direct result of tensile load in the spinal nerves during passive cervical rotation. Concurrently, this shift would increase the distance of the spinal nerve to the contralateral foramen, and thus account for the increased contralateral tensile spinal nerve load observed in several trials. The greater occipital nerve arises from the dorsal ramus of the C2 spinal nerve, emerging between the atlas and axis. It then courses upward, piercing the obliquus capitis inferior, semispinalis capitis, and trapezius muscles, before innervating the skin of the posterior scalp. Therefore, Pan et al.^10^ suggested that the occipital nerve undergoes an increase in tensional load during upper cervical flexion. Although the dissected specimens were prepared to expose the spinal nerves at the foramen level (Fig. 4) to minimize confounding tension from the surrounding tissues, it is still possible that neck flexion during testing influenced the observed tensile load through dural or myofascial tensioning. This potential limitation should be considered when interpreting the results of this study.

Based on our findings and those of previous reports, we propose that the passive flexion rotation test of the cervical spine may provide a useful framework for future clinical validation as a potential indicator of occipital nerve tension. In this position, maximal cervical and subcranial flexion were combined with maximal atlantoaxial rotation, thus loading the occipital nerve. We suggest considering this test positive if the subject’s recognizable symptoms are provoked. Therefore, the potential effects of myodural bridges should be considered in clinical tests. It has been demonstrated that the rectus capitis posterior minor and nuchal ligament have collagenous connections with the dura [15, 25, 32]. Therefore, during upper cervical rotation, elongation of the contralateral rectus capitis posterior minor and nuchal legal ligament is likely to occur, potentially causing contralateral translation of the spinal cord in the spinal canal. This translation, caused by direct dural pulling, leads to increased tensile load on the ipsilateral spinal nerves. Future research should correlate this neural tension test in patients with known occipital neuralgia and occipital nerve blocks in the direction of the atlantoaxial rotation-provoking symptoms.

Nerves of the middle cervical spine (C4–C5) contribute to the brachial plexus. Increased neural tension of the occipital nerves has been associated with conditions such as the carpal tunnel and cervical radiculopathy [1]. This study observed that the changes in the tensile load of the middle cervical spinal nerves (C4–C5) exhibited a more uniform pattern during repeated testing. All trials, except one, displayed a contralateral increase in tensile load in the spinal nerves during passive cervical rotation.

Based on the orientation of the articular facet surfaces, rotation in the mid-cervical spine is coupled with ipsilateral side bending of the neck [33]. Biomechanically, both segmental rotation and side bending result in lateral and posterior translation of the superior articular segment. This translation increases the tensile load on the contralateral spinal nerve in this segment. The findings of this study support the following hypotheses:

Clinically, neural tension of the mid-cervical spine was assessed using upper limb tension tests. Neural tension tests were performed to determine the mechanosensitivity of the median, radial, and ulnar nerves [1, 34]. The validity and reliability of the upper limb tension tests have been demonstrated [35]. The tests were carried out by combining shoulder positioning with elbow, wrist, and hand positioning to increase the tensile load of the respective nerve. At the point of symptom reproduction, the practitioner reduced the joint angle by one until the symptoms disappeared. Next, the subjects were asked to perform a contralateral lateral flexion of the head. A positive test result can cause the symptoms to return [35, 36]. Based on the findings of our study, we suggest that the upper limb tension test be modified to replace lateral neck flexion with rotation. This is a much easier and subject-controlled motion that avoids the necessary flexion to allow for lateral flexion when in the supine position, thus reducing the risk of neural sensitization as a result of the test. Future research should validate this modified upper-limb tension test in vivo.

##  Limitations

This study had several limitations that should be considered when interpreting the findings. The anatomical specimens obtained from the Anatomical Board of the State of Florida were formalin-embalmed cadavers aged >50 years with no available medical history. The absence of clinical data limits our ability to account for pre-existing spinal pathologies, degenerative changes, or neuromusculoskeletal conditions that may influence tissue integrity and biomechanical behavior. Additionally, the natural effects of aging can alter the structural and mechanical properties of neural and connective tissues.

In addition to altering the viscoelastic behavior of neural and connective tissues, formalin embalming may also affect vertebral kinematics by increasing joint stiffness, reducing segmental mobility, and modifying the passive response of surrounding structures. These factors could have influenced the magnitude or pattern of neural tension observed during passive movements [26, 37]. These alterations may have influenced the observed tensile load responses, thereby affecting the external validity of our results. Unlike in vivo conditions, cadaveric models lack active neuromuscular control, which is an important factor for modulating neural tension in living subjects. Consequently, the mechanical behavior of the tissues under study may not fully replicate physiological responses.

Furthermore, the specimens had previously been dissected by students as part of their anatomy training, which may have introduced variability in the quality, depth, and consistency of the dissections. This factor could have affected the intactness and visibility of the cervical nerves, thereby influencing the measurement accuracy and consistency across specimens.

Technical limitations were encountered during experimental procedures. Many upper cervical nerves were damaged during the initial testing, rendering them unsuitable for repeated measurements and reducing the final sample size to five cadavers. This limitation affects the statistical power and generalizability of the findings. Furthermore, the employed force measurement devices were incapable of detecting tensile loads below 1.6 N in the horizontal position. Initial testing with a 10 N baseline load led to ruptures in sensitive neural structures, particularly the C1 and C2 spinal nerves. To prevent further damage, a reduced baseline load of 5 N is applied during the primary testing phase.

Reliability was assessed only at the intraobserver level, as all measurements were performed by a single investigator. Inter-rater and test–retest reliability could not be established due to the cadaveric design and limited availability of intact cervical specimens, which should be considered when interpreting the reproducibility of the findings.

Further in vitro studies with larger and more controlled samples are necessary to confirm and expand upon these findings. In particular, further exploration of the role of mid-cervical myodural bridges and their potential influence on neural tension dynamics could provide valuable insights into the mechanical interplay between the dural and neural structures in the cervical spine. Furthermore, although rotation was standardized by bringing the head to the anatomical end range in each direction, the natural asymmetry between right and left cervical rotation may have influenced the magnitude of the measured tensile loads.

Additionally, anatomical variations in the intervertebral foramen (e.g., osteophytes or scalene insertions) may have influenced nerve positioning and tensile responses, introducing further variability across specimens.

## Conclusions

This exploratory study on five formalin-embalmed cadavers introduced a novel method for quantifying tensile load on the upper and mid-cervical spinal nerves during passive cervical rotation. These results revealed a relationship between cervical rotation and neural tension. An increase in the tensile load was observed on the ipsilateral side of rotation in the upper cervical nerves, particularly involving the greater occipital nerve. These findings suggest that the passive maximal cervical flexion-rotation test may serve as a clinical tool for assessing occipital nerve tension by increasing mechanical strain along the nerve’s anatomical course.

Rotation of the mid-cervical spine is associated with a contralateral increase in tensile load at the cervical spinal nerve at levels C4 and C5. It is proposed that the current upper-limb neurodynamic tests be adapted by substituting the lateral cervical flexion component with controlled cervical rotation, potentially enhancing the sensitivity and specificity in detecting neural mechanosensitivity.

These findings offer insights into the biomechanical interplay between cervical spine kinematics and neural tissue tensioning, supporting the integration of cervical rotation into neural tension-testing protocols. However, given the limitations of cadaveric models, further in vivo studies are required to evaluate the clinical applicability, diagnostic accuracy, and therapeutic implications of these modifications.

## Data Availability

The original contributions presented in the study are included in the article and further inquiries can be directed to the corresponding author.

## Abbreviations

ULLT: Upper limb tension test
ICC: Intraclass correlation coefficient
SEM: Standard error of measurement
MDC: Minimum detectable change
C1: C5–cervical spinal nerve at level 1 to 5
N: Newton (unit of force)
